# Supplementary material for: Exploring the concordance of recommendations across guidelines on chest imaging for the diagnosis and management of COVID-19: A proposed methodological approach based on a case study
Source: PLoS One. 2023 Jul 27;18(7):e0288359. doi: 10.1371/journal.pone.0288359 (PMC10374079; doi:10.1371/journal.pone.0288359)
Supplement: S1 Table — (DOCX) [file pone.0288359.s001.docx]

**S1 Table. Characteristics of related guidelines**

|  | Guideline developing group | Name of the guideline | Language | Type of group | Country/ Region |
| --- | --- | --- | --- | --- | --- |
| [G1]  (1) | British society of Thoracic Imaging | A British Society of Thoracic Imaging statement: considerations in designing local imaging diagnostic algorithms for the COVID-19 pandemic | English | Professional society | UK |
| [G2]  (2) | American College of Radiology | ACR Guidance on COVID-19 and MR Use | English | Professional society | US |
| [G3]  (3) | American College of Radiology | ACR Recommendations for the use of Chest Radiography and Computed Tomography (CT) for Suspected COVID-19 Infection | English | Professional society | US |
| [G4]  (4) | The Royal Australian and New Zealand College of Radiologists | Advice on appropriate use of CT throughout the COVID-19 pandemic | English | Professional society | Australia and New Zealand |
| [G5]  (5) | Hungarian Society of Radiologists | Ajanlasok a mellkasrontgen es a computer tomografia ct hasznalatara feltetelezett covid-19 fertozes esetere | Hungarian | Professional society | Hungary |
| [G6]  (6) | British society of Thoracic Imaging | An update on COVID-19 for the radiologist - A British society of Thoracic Imaging statement | English | Professional society | UK |
| [G7]  (7) | Canadian Society of Thoracic Radiology (CSTR) and Canadian Association of Radiologists (CAR) | Canadian Association of Thoracic Radiology/Canadian Association of Radiologists Consensus Statement Regarding Chest Imaging in Suspected and Confirmed COVID-19 | English | Professional society | Canada |
| [G8]  (8) | Asociacion Colombiana de Infectologia | Consenso colombiano de atención, diagnóstico y manejo de la infección por SARS-COV-2/COVID-19 en establecimientos de atención de la salud | Spanish | Professional society | Colombia |
| [G9]  (9) | European Society of Radiology and European Society of Thoracic Imaging | COVID-19 patients and the Radiology department – advice from the European Society of Radiology (ESR) and the European Society of Thoracic Imaging (ESTI) | English | Professional society | Europe |
| [G10]  (10) | National Institute for Public Health of the Netherlands | Diagnosis, Prevention, and Treatment of Thromboembolic Complications in COVID-19: Report of the National Institute for Public Health of the Netherlands | English | Governmental | Netherlands |
| [G11]  (11) | German Radiological Society | Empfehlungen der AG Thoraxdiagnostik der deutschen Röntgengesellschaft zur klinischen Anwendung der Thoraxbildgebung und strukturierten CT Befundung bei COVID-19 Pandemie | German | Professional society | Germany |
| [G12]  (12) | Société Française De Radiologie | Epidémie de Covid-19 : Point Sur L'imagerie | French | Professional society | France |
| [G13]  (13) | Société Tunisienne de Radiologie and Collège d’Imagerie Médicale, Biophysique et médecine nucléaire | Épidémie de Covid-19 Recommandations pour l’imagerie | French | Professional society | Tunisia |
| [G14]  (14) | Spanish Society of Radiology | Guía básica de indicaciones de pruebas de imagen en la infección COVID-19 | Spanish | Professional society | Spain |
| [G15]  (15) | The Royal Australian and New Zealand College of Radiologists | Guidelines for CT Chest and Chest Radiograph reporting in patients with suspected COVID-19 infection | English | Professional society | Australia and New Zealand |
| [G16]  (16) | Association of Surgeons of Great Britain & Ireland | Intercollegiate General Surgery Guidance on COVID-19 | English | Professional society | UK |
| [G17]  (17) | Russian Federation Health Ministry | ВРЕМЕННЫЕ МЕТОДИЧЕСКИЕ РЕКОМЕНДАЦИИ: ПРОФИЛАКТИКА, ДИАГНОСТИКА И ЛЕЧЕНИЕ НОВОЙ КОРОНАВИРУСНОЙ ИНФЕКЦИИ (COVID-19) | Russian | Governmental | Russia |
| [G18]  (18) | Expert consensus from various organizations: Society for Pediatric Radiology (SPR), European Society of Paediatric Radiology (ESPR), Asian and Oceanic Society for Pediatric Radiology (AOSPR), Sociedad Latino Americana de Radiologica Pediatrica (SLAPR), South African Society of Pediatric Imaging (SASPI), and Radiology Society of the Emirates (RSE) | International Expert Consensus Statement on Chest Imaging in Pediatric COVID-19 Patient Management: Imaging Findings, Imaging Study Reporting and Imaging Study Recommendations | English | Professional society | Various |
| [G19]  (19) | Spanish Society of Radiology | La radiología desde la aparición de la infección COVID-19 | Spanish | Professional society | Spain |
| [G20]  (20) | Oman Ministry of Health | National Clinical Management Pathways For Hospitalized Patients With Covid-19 | English | Governmental | Oman |
| [G21]  (21) | Brazilian Association of Professionals in Infection Control and Hospital Epidemiology | Orientações sobre Diagnóstico, Tratamento e Isolamento de Pacientes com COVID-19 | Portugese | Professional society | Brazil |
| [G22]  (22) | Chinese Society of Radiology, Chinese Medical Association | Radiological diagnosis of COVID 19 : expert recommendation from the Chinese Society of Radiology | English | Professional society | China |
| [G23]  (23) | Radiological Society of North America | Radiological Society of North America Expert Consensus Statement on Reporting Chest CT Findings Related to COVID-19: Interobserver Agreement Between Chest Radiologists | English | Professional society | North America |
| [G24]  (24) | Slovak Radiological Society | Rádiologická Diagnostika V Súvislosti S Ochorením Covid-19 | Slovakian | Professional society | Slovakia |
| [G25]  (25) | The Royal College of Radiologists | RCR position on the role of CT in patients suspected with COVID-19 infection | English | Professional society | UK |
| [G26]  (26) | Colégio Brasileiro de Radiologia | Recomendações de uso de métodos de imagem para pacientes suspeitos de infecção pelo COVID-19 | Portugese | Professional society | Brazil |
| [G27]  (27) | Indian Radiological and Imaging Association | Recommendations of IRIA ICRI chest subspecialty group | English | Professional society | India |
| [G28]  (28) | Haute Autorité de Santé | Réponses rapides dans le cadre du COVID-19 - Indications du scanner thoracique | French | Governmental | France |
| [G29]  (29) | Canadian Society of Thoracic Radiology (CSTR) and Canadian Association of Radiologists (CAR) | The Canadian Association of Radiologists (CAR) and the Canadian Society on Thoracic Radiology (CSTR) Recommendations on COVID-19 Management in Imaging Departments | English | Professional society | Canada |
| [G30]  (30) | Canadian Association for Interventional Radiology (CAIR) and Canadian Association of Radiologists (CAR) | The Canadian Society of Thoracic Radiology (CSTR) and Canadian Association of Radiologists (CAR) Consensus Statement Regarding Chest Imaging in Suspected and Confirmed COVID-19 | English | Professional society | Canada |
| [G31]  (31) | British Society of Thoracic Imaging (BSTI) | The continuing evolution of COVID-19 imaging pathways in the UK: a British Society of Thoracic Imaging expert reference group update | English | Professional society | UK |
| [G32]  (32) | Fleischner Society | The Role of Chest Imaging in Patient Management during the COVID-19 Pandemic: A Multinational Consensus Statement from the Fleischner Society | English | Professional society | US |

**References**

1. Nair A, Rodrigues JCL, Hare S, Edey A, Devaraj A, Jacob J, et al. A British Society of Thoracic Imaging statement: considerations in designing local imaging diagnostic algorithms for the COVID-19 pandemic. Clin Radiol. 2020;75(5):329-34.

2. American College of Radiology. ACR Guidance on COVID-19 and MR Use 2020 [cited 2021]. Available from: https://[www.acr.org/Clinical-Resources/Radiology-Safety/MR-Safety/COVID-19-and-MR-Use](http://www.acr.org/Clinical-Resources/Radiology-Safety/MR-Safety/COVID-19-and-MR-Use).

3. American College of Radiology. ACR Recommendations for the use of Chest Radiography and Computed Tomography (CT) for Suspected COVID-19 Infection 2020 [cited 2021]. Available from: https://[www.acr.org/Advocacy-and-Economics/ACR-Position-Statements/Recommendations-for-Chest-Radiography-and-CT-for-Suspected-COVID19-Infection](http://www.acr.org/Advocacy-and-Economics/ACR-Position-Statements/Recommendations-for-Chest-Radiography-and-CT-for-Suspected-COVID19-Infection).

4. The Royal Australian and New Zealand College of Radiologists. Advice on appropriate use of CT throughout the COVID-19 pandemic 2020 [cited 2021]. Available from: https://[www.ranzcr.com/college/document-library/advice-on-appropriate-use-of-ct-throughout-the-covid-19-pandemic](http://www.ranzcr.com/college/document-library/advice-on-appropriate-use-of-ct-throughout-the-covid-19-pandemic).

5. Hungarian Society of Radiologists. Ajanlasok a mellkasrontgen es a computer tomografia ct hasznalatara feltetelezett covid-19 fertozes esetere 2020 [cited 2021]. Available from: https://[www.doki.net/tarsasag/radiologia/hirek.aspx?nid=99051](http://www.doki.net/tarsasag/radiologia/hirek.aspx?nid=99051).

6. Rodrigues JCL, Hare SS, Edey A, Devaraj A, Jacob J, Johnstone A, et al. An update on COVID-19 for the radiologist - A British society of Thoracic Imaging statement. Clin Radiol. 2020;75(5):323-5.

7. Dennie C, Hague C, Lim RS, Manos D, Memauri BF, Nguyen ET, et al. Canadian Society of Thoracic Radiology/Canadian Association of Radiologists Consensus Statement Regarding Chest Imaging in Suspected and Confirmed COVID-19. Canadian Association of Radiologists Journal. 2020;71(4):470-81.

8. Carlos Humberto Saavedra Trujillo. Consenso colombiano de atención, diagnóstico y manejo de la infección por SARS-COV-2/COVID-19 en establecimientos de atención de la salud 2020 [cited 2021]. Available from: https://[www.revistainfectio.org/index.php/infectio/article/view/851/0](http://www.revistainfectio.org/index.php/infectio/article/view/851/0).

9. Revel MP, Parkar AP, Prosch H, Silva M, Sverzellati N, Gleeson F, et al. COVID-19 patients and the radiology department - advice from the European Society of Radiology (ESR) and the European Society of Thoracic Imaging (ESTI). Eur Radiol. 2020;30(9):4903-9.

10. Oudkerk M, Büller HR, Kuijpers D, van Es N, Oudkerk SF, McLoud T, et al. Diagnosis, Prevention, and Treatment of Thromboembolic Complications in COVID-19: Report of the National Institute for Public Health of the Netherlands. Radiology. 2020;297(1):E216-e22.

11. Jens Vogel-Claussen JL-Z, Prerana Agarwal, Jürgen Biederer, Hans-Ulrich Kauczor, Sebastian Ley, Hilmar Kühl. Empfehlungen der AG Thoraxdiagnostik der Deutschen Röntgengesellschaft zur klinischen Anwendung der Thoraxbildgebung und strukturierten CT-Befundung bei COVID-19-Pandemie 2020 [cited 2021]. Available from: https://eref.thieme.de/ejournals/reader/1438-9010_2020_07/10.1055-a-1174-8378.

12. Société Française De Radiologie. Epidémie de Covid-19 : Point Sur L'imagerie 2020 [cited 2021]. Available from: https://ebulletin.radiologie.fr/actualites-covid-19/epidemie-covid-19-point-limagerie.

13. Société Tunisienne de Radiologie and Collège d’Imagerie Médicale Bemn. Épidémie de Covid-19 Recommandations pour l’imagerie 2020 [cited 2021]. Available from: <http://strtn.org/media/articles/Proc%C3%A9dures%20%20pour%20l'imagerie%20covid%20%20STR%20INEAS.pdf>.

14. Spanish Society of Radiology. Guía básica de indicaciones de pruebas de imagen en la infección COVID-19 2020 [cited 2021]. Available from: https://seram.es/images/site/Recomendaciones_imagen_SERAM_COVID_19.pdf.

15. The Royal Australian and New Zealand College of Radiologists. Guidelines for CT Chest and Chest Radiograph reporting in patients with suspected COVID-19 infection 2020 [cited 2021]. Available from: https://[www.ranzcr.com/college/document-library/guidelines-for-ct-chest-and-chest-radiograph-reporting-in-patients-with-suspected-covid-19-infection](http://www.ranzcr.com/college/document-library/guidelines-for-ct-chest-and-chest-radiograph-reporting-in-patients-with-suspected-covid-19-infection).

16. Association of Surgeons of Great Britain & Ireland. Intercollegiate General Surgery Guidance on COVID-19 2020. Available from: https://[www.rcsed.ac.uk/news-public-affairs/news/2020/march/intercollegiate-general-surgery-guidance-on-covid-19-update](http://www.rcsed.ac.uk/news-public-affairs/news/2020/march/intercollegiate-general-surgery-guidance-on-covid-19-update).

17. Russian Federation Health Ministry. ВРЕМЕННЫЕ МЕТОДИЧЕСКИЕ РЕКОМЕНДАЦИИ: ПРОФИЛАКТИКА, ДИАГНОСТИКА И ЛЕЧЕНИЕ НОВОЙ КОРОНАВИРУСНОЙ ИНФЕКЦИИ (COVID-19) 2020 [cited 2020].

18. Foust AM, Phillips GS, Chu WC, Daltro P, Das KM, Garcia-Peña P, et al. International Expert Consensus Statement on Chest Imaging in Pediatric COVID-19 Patient Management: Imaging Findings, Imaging Study Reporting, and Imaging Study Recommendations. Radiology Cardiothoracic imaging. 2020;2(2):e200214.

19. Spanish Society of Radiology. La radiología desde la aparición de la infección COVID-19 2020 [cited 2021]. Available from: https://[www.seram.es/images/site/Futuro_Radiologia_COVID_SERAM_2.pdf](http://www.seram.es/images/site/Futuro_Radiologia_COVID_SERAM_2.pdf).

20. Oman Ministry of Health. National Clinical Management Pathways For Hospitalized Patients With Covid-19 2020. Available from: NA.

21. Viviane Maria de Carvalho Hessel Dias CAdC, Cláudia Fernanda de Lacerda Vidal, Mirian Freitas Dal Ben Corradi, Lessandra Michelin, Valdair Muglia, Jaime Luis Lopes Rocha, Sílvia Figueiredo Costa, Priscila Rosalba Domingos de Oliveira, Cláudia Maio Carrilho, Alberto Chebabo, Rogean Rodrigues Nunes, Luis Antonio dos Santos Diego, Alair Sarmet Santos, Marcelo Carneiro, Arthur Soares Souza Junior, Dante Escuissato, Cesar Araújo Neto, Luis Fernando Waib, Ricardo Martins, Rosemeri Maurici, José Miguel Chatkin, Denise Brandão, Suzana Margareth Ajeje Lobo, Mirella Cristine de Oliveira, Jairo Silva Alves, Marcelo Mazza Nascimento, José A. Moura-Neto. Orientações sobre Diagnóstico, Tratamento e Isolamento de Pacientes com COVID-19 2020. Available from: https://jic-abih.com.br/index.php/jic/article/view/295.

22. Chinese Society of Radiology and Chinese Medical Association. Radiological diagnosis of COVID 19 : expert recommendation from the Chinese Society of Radiology 2020. Available from: NA.

23. Byrne D, Neill SBO, Müller NL, Müller CIS, Walsh JP, Jalal S, et al. RSNA Expert Consensus Statement on Reporting Chest CT Findings Related to COVID-19: Interobserver Agreement Between Chest Radiologists. Canadian Association of Radiologists journal = Journal l'Association canadienne des radiologistes. 2021;72(1):159-66.

24. Slovak Radiological Society. Rádiologická Diagnostika V Súvislosti S Ochorením Covid-19 2020. Available from: https://[www.slovakradiology.sk/sk/rAdiologickA-diagnostika-v-sUvislosti-s-ochorenIm-covid-19-1/](http://www.slovakradiology.sk/sk/rAdiologickA-diagnostika-v-sUvislosti-s-ochorenIm-covid-19-1/).

25. The Royal College of Radiologists. The role of CT in patients suspected with COVID-19 infection 2020. Available from: https://[www.rcr.ac.uk/posts/role-ct-patients-suspected-covid-19-infection-12-march-2020](http://www.rcr.ac.uk/posts/role-ct-patients-suspected-covid-19-infection-12-march-2020).

26. Colégio Brasileiro de Radiologia. Recomendações de uso de métodos de imagem para pacientes suspeitos de infecção pelo COVID-19 2020. Available from: https://cbr.org.br/wp-content/uploads/2020/03/CBR_Recomenda%C3%A7%C3%B5es-de-uso-de-m%C3%A9todos-de-imagem.pdf.

27. Indian Radiological and Imaging Association. Recommendations of IRIA ICRI chest subspecialty group 2020. Available from: <http://irp-cdn.multiscreensite.com/e482ea7d/files/uploaded/IRIA_ICRI_guidlines_on_Imaging_in_COVID-19_Patients.pdf>.

28. Haute Autorité de Santé. Réponses rapides dans le cadre du COVID-19 - Indications du scanner thoracique 2020. Available from: https://webzine.has-sante.fr/upload/docs/application/pdf/2020-04/reponse_rapide_codid-19_indication_tdm_mel2.pdf.

29. Canadian Society of Thoracic Radiology (CSTR) and Canadian Association of Radiologists (CAR). The Canadian Association of Radiologists (CAR) and the Canadian Society on Thoracic Radiology (CSTR) Recommendations on COVID-19 Management in Imaging Departments 2020.

30. Canadian Association for Interventional Radiology (CAIR) and Canadian Association of Radiologists (CAR). The Canadian Society of Thoracic Radiology (CSTR) and Canadian Association of Radiologists (CAR) Consensus Statement Regarding Chest Imaging in Suspected and Confirmed COVID-19 2020.

31. Hare SS, Rodrigues JCL, Nair A, Jacob J, Upile S, Johnstone A, et al. The continuing evolution of COVID-19 imaging pathways in the UK: a British Society of Thoracic Imaging expert reference group update. Clin Radiol. 2020;75(6):399-404.

32. Rubin GD, Ryerson CJ, Haramati LB, Sverzellati N, Kanne JP, Raoof S, et al. The Role of Chest Imaging in Patient Management During the COVID-19 Pandemic: A Multinational Consensus Statement From the Fleischner Society. Chest. 2020;158(1):106-16.
