# Supplementary material for: Exploring the concordance of recommendations across guidelines on chest imaging for the diagnosis and management of COVID-19: A proposed methodological approach based on a case study
Source: PLoS One. 2023 Jul 27;18(7):e0288359. doi: 10.1371/journal.pone.0288359 (PMC10374079; doi:10.1371/journal.pone.0288359)
Supplement: S2 Table — Blue: reference recommendation, Green: entirely matching, Light yellow: partly matching–less specific, and Dark yellow: partly matching–more specific. (DOCX) [file pone.0288359.s002.docx]

**S2 Table. Results of the construction of the PICOs and matching of related recommendations for each of the reference recommendations.**

Abbreviations: CT: computed tomography, CXR: chest X-ray

**Legend:**

| Reference recommendation |
| --- |
| Entirely matching |
| Partly matching - Less specific |
| Partly matching - More specific |

**Reference PICO 1**

| **Guideline** | **Related recommendation** | **Population** | **Intervention** | **Comparator** | **Setting** | **Purpose** |
| --- | --- | --- | --- | --- | --- | --- |
| WHO | **For asymptomatic contacts of patients with COVID-19, WHO suggests not using chest imaging for the diagnosis of COVID-19.** | Asymptomatic contacts of patients with COVID-19 | Chest imaging (including chest X-Ray, CT scan, lung US) | Alternative chest imaging (including chest X-Ray, CT scan, lung US) OR No chest imaging |  |  |
| **[G32]** | Imaging is not routinely indicated as a screening test for COVID-19 in asymptomatic individuals. | asymptomatic individuals | Imaging | no imaging |  |  |
| **[G18]** | Imaging is not generally indicated as a screening tool for symptomatic or asymptomatic pediatric patients with suspected COVID-19 infection. | symptomatic or asymptomatic pediatric patients with suspected COVID-19 infection* | Imaging | no imaging |  |  |
| **[G26]** | In patients with suspected COVID-19 infection, and are asymptomatic, it is not advised to perform any imaging exam. | patients with suspected COVID-19 infection, and are asymptomatic | Imaging | no imaging |  |  |
| **[G24]** | In asymptomatic patients with suspected COVID-19 infection due to close contact with a patient with confirmed COVID-19 infection, no imaging exam (no CT, no CXR) is recommended. | asymptomatic patients with suspected COVID-19 infection due to close contact with a patient with confirmed COVID-19 infection | imaging exam (CT, CXR) | no imaging exam (no CT, no CXR) |  |  |
| **[G9]** | In patients with mild or no symptoms, CT should not be performed as a screening test. | patients with mild or no symptoms | CT scan | no CT scan |  |  |
| **[G1]** | Patients with high clinical suspicion and asymptomatic, we cannot envisage the practical value of using CT. | Patients with high clinical suspicion and asymptomatic | CT scan | no CT scan |  |  |
| **[G19]** | Imaging tests are not indicated for COVID screening in asymptomatic patients. | asymptomatic patients | Imaging | no imaging |  |  |

***** We judged the population to be less specific than that of the reference population since it includes asymptomatic and symptomatic patients, although the pediatric population is more specific.

**Reference PICO 2.1**

| **Guideline** | **Related recommendation** | **Population** | **Intervention** | **Comparator** | **Setting** | **Purpose** |
| --- | --- | --- | --- | --- | --- | --- |
| **WHO** | **For symptomatic patients with suspected COVID-19, WHO suggests not using chest imaging for the diagnostic workup of COVID-19 when RT-PCR testing is available with timely results.** | Symptomatic patients with suspected COVID-19 | Chest imaging (including chest X-Ray, CT scan, lung US) | Alternative chest imaging (including chest X-Ray, CT scan, lung US) OR No chest imaging | RT-PCR testing is available with timely results |  |
| **[G25]** | In patients with suspected COVID-19 infection, there is no current role for CT in the diagnostic assessment of these patients. | patients with suspected COVID-19 infection | CT scan | no CT scan |  |  |
| **[G3]** | In patients with suspected COVID-19 infection, CT scan should not be used to screen for or as a first-line test for diagnosis. | patients with suspected COVID-19 infection | CT scan | no CT scan |  |  |
| **[G18]** | Imaging is not generally indicated as a screening tool for symptomatic or asymptomatic pediatric patients with suspected COVID-19 infection | symptomatic or asymptomatic pediatric patients with suspected COVID-19 infection* | chest imaging | no chest imaging |  |  |
| **[G26]** | In patients with suspected COVID-19 infection, high-resolution computed tomography (HRCT) of the chest should not be used separately for the diagnosis of COVID-19, nor should it be performed for disease tracking. | patients with suspected COVID-19 infection | high-resolution chest CT | no high-resolution chest CT |  |  |
| **[G9]** | In patients with mild or no symptoms, CT should not be performed as a screening test. | patients with mild or no symptoms | CT scan | no CT scan |  |  |
| **[G5]** | In patients with suspected or confirmed COVID-19, X-ray or CT is not currently recommended for the diagnosis of COVID-19. | suspected or confirmed COVID-19 | X-ray or CT scan | no X-ray or CT scan |  |  |
| **[G12]** | There is currently no indication to perform a chest CT scan for the purpose of screening in patients without signs of severity and without comorbidities. | patients without signs of severity and without comorbidities | chest CT | no chest CT |  |  |
| **[G30]** | In patients with suspected or confirmed COVID-19 patients, CT should not be used by default to diagnose COVID-19 in patients. | patients with suspected or confirmed COVID-19 | CT scan | no CT scan |  |  |
| **[G30]** | A chest x-ray is not recommended for people with mild symptoms. | patients with mild symptoms | chest x-ray | no chest x-ray |  |  |
| **[G28]** | Do not perform a chest scan for screening purposes on patients without signs of severity for the diagnosis of COVID-19 | patients without signs of severity | CT scan | no CT scan |  |  |
| **[G17]** | In the presence of symptoms and clinical signs of SARS, and there is a suspicion of COVID-19 (including on the basis of anamnestic data): - Chest CT is recommended; - If CT scanning cannot be performed, then chest X-ray and ultrasound examinations are recommended. | patients with symptoms and clinical signs of SARS, and there is a suspicion of COVID-19 (including on the basis of anamnestic data) | Imaging | no imaging |  |  |

***** We judged the population to be less specific than that of the reference population since it includes asymptomatic and symptomatic patients, although the pediatric population is more specific.

**Reference PICO 2.2**

| **Guideline** | **Related recommendation** | **Population** | **Intervention** | **Comparator** | **Setting** | **Purpose** |
| --- | --- | --- | --- | --- | --- | --- |
| **WHO** | **For symptomatic patients with suspected COVID-19, WHO suggests using chest imaging for the diagnostic workup of COVID-19 when: (1) RT-PCR testing is not available; (2) RT-PCR testing is available, but results are delayed; and (3) initial RT-PCR testing is negative, but with high clinical suspicion of COVID-19.** | Symptomatic patients with suspected COVID-19 | Chest imaging (including chest X-Ray, CT scan, lung US) | Alternative chest imaging (including chest X-Ray, CT scan, lung US) OR No chest imaging | where laboratory testing (RT PCR) is not available/results are delayed/results are initially negative or need for early recognition |  |
| **[G25]** | In patients with suspected COVID-19 infection, there is no current role for CT in the diagnostic assessment of these patients. | patients with suspected COVID-19 infection | CT scan | no CT scan |  |  |
| **[G3]** | In patients with suspected COVID-19 infection, CT scan should not be used to screen for or as a first-line test for diagnosis. | patients with suspected COVID-19 infection | CT scan | no CT scan |  |  |
| **[G18]** | Imaging is not generally indicated as a screening tool for symptomatic or asymptomatic pediatric patients with suspected COVID-19 infection | symptomatic or asymptomatic pediatric patients with suspected COVID-19 infection* | chest imaging | no chest imaging |  |  |
| **[G26]** | In patients with suspected COVID-19 infection, high-resolution computed tomography (HRCT) of the chest should not be used separately for the diagnosis of COVID-19, nor should it be performed for disease tracking. | patients with suspected COVID-19 infection | high-resolution chest CT | no high-resolution chest CT |  |  |
| **[G24]** | In patients with clinical suspicion of COVID-19 infection, and cannot be tested (e.g. due to insufficient number of tests) and have received recommendations for home quarantine, no imaging exam is indicated. | patients with clinical suspicion of COVID-19 infection and received recommendations for home quarantine | chest imaging | no chest imaging | cannot be tested (e.g. due to insufficient number of tests) |  |
| **[G9]** | In patients with mild or no symptoms, CT should not be performed as a screening test. | patients with mild or no symptoms | CT scan | no CT scan |  |  |
| **[G5]** | In patients with suspected or confirmed COVID-19, X-ray or CT is not currently recommended for the diagnosis of COVID-19. | suspected or confirmed COVID-19 | X-ray or CT scan | no X-ray or CT scan |  |  |
| **[G12]** | There is currently no indication to perform a chest CT scan for the purpose of screening in patients without signs of severity and without comorbidities. | patients without signs of severity and without comorbidities | chest CT | no chest CT |  |  |
| **[G30]** | In patients with suspected or confirmed COVID-19 patients, CT should not be used by default to diagnose COVID-19 in patients. | patients with suspected or confirmed COVID-19 | CT scan | no CT scan |  |  |
| **[G30]** | A chest x-ray is not recommended for people with mild symptoms. | patients with mild symptoms | chest x-ray | no chest x-ray |  |  |
| **[G28]** | Do not perform a chest scan for screening purposes on patients without signs of severity for the diagnosis of COVID-19 | patients without signs of severity | CT scan | no CT scan |  |  |
| **[G17]** | In the presence of symptoms and clinical signs of SARS, and there is a suspicion of COVID-19 (including on the basis of anamnestic data): - Chest CT is recommended; - If CT scanning cannot be performed, then chest X-ray and ultrasound examinations are recommended. | patients with symptoms and clinical signs of SARS, and there is a suspicion of COVID-19 (including on the basis of anamnestic data) | Imaging | no imaging |  |  |
| **[G1]** | In the minority of patients with high clinical suspicion but negative initial RT-PCR, we cannot envisage the practical value of using CT. | patients with high clinical suspicion but negative initial RT-PCR | CT scan | no CT scan |  |  |

**Reference PICO 3**

| **Guideline** | **Related recommendation** | **Population** | **Intervention** | **Comparator** | **Setting** | **Purpose** |
| --- | --- | --- | --- | --- | --- | --- |
| **WHO** | For patients with suspected or confirmed COVID-19, not currently hospitalized and with mild symptoms, WHO suggests using chest imaging in addition to clinical and laboratory assessment to decide on hospital admission versus home discharge. | patients with suspected or confirmed COVID-19, not currently hospitalized and with mild symptoms | chest imaging in addition to clinical and laboratory assessment | clinical and laboratory assessment |  | to decide on hospital admission versus home discharge |
| **[G32]** | In patients with mild features (at an outpatient clinic or via telehealth) consistent with COVID-19, any pre-test probability, no significant resource constraint, and positive COVID-19 test, imaging is indicated in patients with risk factors for disease progression. | patients with mild features (at an outpatient clinic or via telehealth) consistent with COVID-19, any pre-test probability, no significant resource constraint, and positive COVID-19 test and with risk factors for disease progression | imaging | no imaging |  |  |
| **[G18]** | Imaging is not indicated for a pediatric patient presenting with mild clinical symptoms unless the patient has risk factors for disease progression or develops worsening clinical symptoms. | pediatric patient presenting with mild clinical symptoms | imaging | no imaging |  |  |
| **[G18]** | Imaging is indicated for a pediatric patient presenting with mild clinical symptoms and has risk factors for disease progression or develops worsening clinical symptoms. | pediatric patient presenting with mild clinical symptoms and risk factors for disease progression or develops worsening clinical symptoms | imaging | no imaging |  |  |
| **[G26]** | In patients with confirmed COVID-19 infection (positive PCR/Anti-IgM), with mild-to-moderate symptoms, any imaging exam is recommended. | patients with confirmed COVID-19 infection (positive PCR/Anti-IgM), with mild-to-moderate symptoms, | imaging | no imaging |  |  |
| **[G1]** | In patients with COVID-19 RT-PCR positive result, BSTI does not envisage a role for CT in this setting. | patients with COVID-19 RT-PCR positive result | chest CT | no chest CT |  |  |
| **[G13]** | Pauci symptomatic patients, without comorbidities and not hospitalized, do not perform CT imaging | Pauci symptomatic patients, without comorbidities, not hospitalized | chest CT | no chest CT |  |  |
| **[G21]** | For mild/moderate symptomatic patients with positive PCR/Anti-IgM, no imaging exam is recommended. | mild/moderate symptomatic patients with positive PCR/Anti-IgM | imaging | no imaging |  |  |

**Reference PICO 4**

| **Guideline** | **Related recommendation** | **Population** | **Intervention** | **Comparator** | **Setting** | **Purpose** |
| --- | --- | --- | --- | --- | --- | --- |
| **WHO** | For patients with suspected or confirmed COVID-19, not currently hospitalized and with moderate to severe symptoms, WHO suggests using chest imaging in addition to clinical and laboratory assessment to decide on regular ward admission versus intensive care unit (ICU) admission. | patients with suspected or confirmed COVID-19, not currently hospitalized and with moderate to severe symptoms | chest imaging in addition to clinical and laboratory assessment | clinical and laboratory assessment |  | to decide on regular ward admission versus intensive care unit (ICU) admission |
| **[G32]** | Imaging is indicated for patients with moderate to severe features of COVID-19 regardless of COVID-19 test results. | patients with moderate to severe regardless of COVID-19 test results | imaging | no imaging |  |  |
| **[G1]** | In patients with COVID-19 RT-PCR positive result, BSTI does not envisage a role for CT in this setting. | patients with COVID-19 RT-PCR positive result | chest CT | no chest CT |  |  |
| **[G21]** | For mild/moderate symptomatic patients with positive PCR/Anti-IgM, no imaging exam is recommended. | mild/moderate symptomatic patients with positive PCR/Anti-Ig | imaging | no imaging |  |  |

**Reference PICO 5**

| **Guideline** | **Related recommendation** | **Population** | **Intervention** | **Comparator** | **Setting** | **Purpose** |
| --- | --- | --- | --- | --- | --- | --- |
| **WHO** | For patients with suspected or confirmed COVID-19, currently hospitalized and with moderate to severe symptoms, WHO suggests using chest imaging in addition to clinical and laboratory assessment to inform therapeutic management. | patients with suspected or confirmed COVID-19, currently hospitalized and moderate or severe symptoms | chest imaging in addition to clinical and laboratory assessment | No chest imaging |  |  |
| **[G18]** | In pediatric patients with moderate-to-severe COVID-19, sequential CXRs are appropriate as clinically needed basis. | pediatric patients with moderate-to-severe COVID-19 | sequential chest X-ray | no sequential chest X-ray |  |  |
| **[G24]** | In patients with COVID-19 infection, who develop clinical complications, chest CT imaging is the primary imaging technique in patients who are able to be transported to radiological workplace. | patients with COVID-19 infection, who develop clinical complications who are able to be transported to radiological workplace | chest CT | no chest CT |  |  |
| **[G12]** | The realization of a thoracic scanner without injection in fine sections is currently indicated in patients with a suspected or confirmed diagnosis and initial or secondary signs of clinical severity (dyspnea, desaturation, etc.) under hospital care | suspected or confirmed diagnosis and initial or secondary signs of clinical severity (dyspnea, desaturation, etc.) under hospital care | chest CT without injection | no chest CT without injection |  |  |
