# Supplementary material for: Exploring the concordance of recommendations across guidelines on chest imaging for the diagnosis and management of COVID-19: A proposed methodological approach based on a case study
Source: PLoS One. 2023 Jul 27;18(7):e0288359. doi: 10.1371/journal.pone.0288359 (PMC10374079; doi:10.1371/journal.pone.0288359)
Supplement: S3 Table — Grey: reference recommendation, Red: strongly against, Red: conditional against, Light green: conditional for, and Dark green: strongly for. (DOCX) [file pone.0288359.s003.docx]

**S3 Table. Concordance of the matching recommendations and the reference recommendations in terms of direction and strength**

Legend:

| **Reference recommendation** | **Strongly against** | **Conditional against** | **Conditional for** | **Strongly for** |
| --- | --- | --- | --- | --- |

| **Guideline-producing group** | **Recommendation** | **Strongly against** | **Conditional against** | **Conditional for** | **Strongly for** |
| --- | --- | --- | --- | --- | --- |
| **WHO R1** | **For asymptomatic contacts of patients with COVID-19, WHO suggests not using chest imaging for the diagnosis of COVID-19.** |  |  |  |  |
| **[G32]** | Imaging is not routinely indicated as a screening test for COVID-19 in asymptomatic individuals. |  |  |  |  |
| **[G18]** | Imaging is not generally indicated as a screening tool for symptomatic or asymptomatic pediatric patients with suspected COVID-19 infection. |  |  |  |  |
| **[G26]** | In patients with suspected COVID-19 infection, and are asymptomatic, it is not advised to perform any imaging exam. |  |  |  |  |
| **[G24]** | In asymptomatic patients with suspected COVID-19 infection due to close contact with a patient with confirmed COVID-19 infection, no imaging exam (no CT, no CXR) is recommended. |  |  |  |  |
| **[G9]** | In patients with mild or no symptoms, CT should not be performed as a screening test. |  |  |  |  |
| **[G1]** | Patients with high clinical suspicion and asymptomatic, we cannot envisage the practical value of using CT. |  |  |  |  |
| **[G19]** | Imaging tests are not indicated for COVID screening in asymptomatic patients. |  |  |  |  |
| **WHO R2.1** | **For symptomatic patients with suspected COVID-19, WHO suggests not using chest imaging for the diagnostic workup of COVID-19 when RT-PCR testing is available with timely results.** |  |  |  |  |
| **[G25]** | In patients with suspected COVID-19 infection, there is no current role for CT in the diagnostic assessment of these patients. |  |  |  |  |
| **[G3]** | In patients with suspected COVID-19 infection, CT scan should not be used to screen for or as a first-line test for diagnosis. |  |  |  |  |
| **[G18]** | Imaging is not generally indicated as a screening tool for symptomatic or asymptomatic pediatric patients with suspected COVID-19 infection |  |  |  |  |
| **[G26]** | In patients with suspected COVID-19 infection, high-resolution computed tomography (HRCT) of the chest should not be used separately for the diagnosis of COVID-19, nor should it be performed for disease tracking. |  |  |  |  |
| **[G9]** | In patients with mild or no symptoms, CT should not be performed as a screening test. |  |  |  |  |
| **[G5]** | In patients with suspected or confirmed COVID-19, X-ray or CT is not currently recommended for the diagnosis of COVID-19. |  |  |  |  |
| **[G12]** | There is currently no indication to perform a chest CT scan for the purpose of screening in patients without signs of severity and without comorbidities. |  |  |  |  |
| **[G30]** | In patients with suspected or confirmed COVID-19 patients, CT should not be used by default to diagnose COVID-19 in patients. |  |  |  |  |
| **[G30]** | A chest x-ray is not recommended for people with mild symptoms. |  |  |  |  |
| **[G28]** | Do not perform a chest scanner for screening purposes on patients without signs of severity for the diagnosis of COVID-19. |  |  |  |  |
| **[G17]** | In the presence of symptoms and clinical signs of SARS, and there is a suspicion of COVID-19 (including on the basis of anamnestic data): - Chest CT is recommended; - If CT scanning cannot be performed, then chest X-ray and ultrasound examinations are recommended. |  |  |  |  |
| **WHO R2.2** | **For symptomatic patients with suspected COVID-19, WHO suggests not using chest imaging for the diagnostic workup of COVID-19 when RT-PCR testing is available with timely results.** |  |  |  |  |
| **[G25]** | In patients with suspected COVID-19 infection, there is no current role for CT in the diagnostic assessment of these patients. |  |  |  |  |
| **[G3]** | In patients with suspected COVID-19 infection, CT scan should not be used to screen for or as a first-line test for diagnosis. |  |  |  |  |
| **[G18]** | Imaging is not generally indicated as a screening tool for symptomatic or asymptomatic pediatric patients with suspected COVID-19 infection |  |  |  |  |
| **[G26]** | In patients with suspected COVID-19 infection, high-resolution computed tomography (HRCT) of the chest should not be used separately for the diagnosis of COVID-19, nor should it be performed for disease tracking. |  |  |  |  |
| **[G24]** | In patients with clinical suspecion of COVID-19 infection, and cannot be tested (e.g. due to insufficient number of tests) and have received recommendations for home quarantine, no imaging exam is indicated. |  |  |  |  |
| **[G9]** | In patients with mild or no symptoms, CT should not be performed as a screening test. |  |  |  |  |
| **[G5]** | In patients with suspected or confirmed COVID-19, X-ray or CT is not currently recommended for the diagnosis of COVID-19. |  |  |  |  |
| **[G12]** | There is currently no indication to perform a chest CT scan for the purpose of screening in patients without signs of severity and without comorbidities. |  |  |  |  |
| **[G30]** | In patients with suspected or confirmed COVID-19 patients, CT should not be used by default to diagnose COVID-19 in patients. |  |  |  |  |
| **[G30]** | A chest x-ray is not recommended for people with mild symptoms. |  |  |  |  |
| **[G28]** | Do not perform a chest scanner for screening purposes on patients without signs of severity for the diagnosis of COVID-19. |  |  |  |  |
| **[G17]** | In the presence of symptoms and clinical signs of SARS, and there is a suspicion of COVID-19 (including on the basis of anamnestic data): - Chest CT is recommended; - If CT scanning cannot be performed, then chest X-ray and ultrasound examinations are recommended. |  |  |  |  |
| **[G1]** | In the minority of patients with high clinical suspicion but negative initial RT-PCR, we cannot envisage the practical value of using CT. |  |  |  |  |
| **WHO R3** | **For patients with suspected or confirmed COVID-19, not currently hospitalized and with mild symptoms, WHO suggests using chest imaging in addition to clinical and laboratory assessment to decide on hospital admission versus home discharge.** |  |  |  |  |
| **[G32]** | In patients with mild features (at an outpatient clinic or via telehealth) consistent with COVID-19, any pre-test probability, no significant resource constraint, and positive COVID-19 test, imaging is indicated in patients with risk factors for disease progression. |  |  |  |  |
| **[G18]** | Imaging is not indicated for a pediatric patient presenting with mild clinical symptoms **unless** the patient has risk factors for disease progression or develops worsening clinical symptoms. |  |  |  |  |
| **[G18]** | Imaging is indicated for a pediatric patient presenting with mild clinical symptoms and has risk factors for disease progression or develops worsening clinical symptoms. |  |  |  |  |
| **[G26]** | In patients with confirmed COVID-19 infection (positive PCR/Anti-IgM), with mild-to-moderate symptoms, any imaging exam is recommended. |  |  |  |  |
| **[G1]** | In patients with COVID-19 RT-PCR positive result, BSTI does not envisage a role for CT in this setting. |  |  |  |  |
| **[G13]** | Pauci symptomatic patients, without comorbidities and not hospitalized, do not perform CT imaging |  |  |  |  |
| **[G21]** | For mild/moderate symptomatic patients with positive PCR/Anti-IgM, no imaging exam is recommended. |  |  |  |  |
| **WHO R4** | **For patients with suspected or confirmed COVID-19, not currently hospitalized and with moderate to severe symptoms, WHO suggests using chest imaging in addition to clinical and laboratory assessment to decide on regular ward admission versus intensive care unit (ICU) admission.** |  |  |  |  |
| **[G32]** | Imaging is indicated for patients with moderate to severe features of COVID-19 regardless of COVID-19 test results. |  |  |  |  |
| **[G1]** | In patients with COVID-19 RT-PCR positive result, BSTI does not envisage a role for CT in this setting. |  |  |  |  |
| **[G21]** | For mild/moderate symptomatic patients with positive PCR/Anti-IgM, no imaging exam is recommended. |  |  |  |  |
| **WHO R5** | **For patients with suspected or confirmed COVID-19, currently hospitalized and with moderate to severe symptoms, WHO suggests using chest imaging in addition to clinical and laboratory assessment to inform therapeutic management.** |  |  |  |  |
| **[G18]** | In pediatric patients with moderate-to-severe COVID-19, sequential CXRs are appropriate as clinically needed basis to monitor response to supportive measures, assess clinical deterioration, or evaluate positioning of life support devices. |  |  |  |  |
| **[G24]** | In patients with COVID-19 infection, who develop clinical complications, chest CT imaging is the primary imaging technique in patients who are able to be transported to radiological workplace. |  |  |  |  |
| **[G12]** | The realization of a thoracic scanner without injection in fine sections is currently indicated in patients with a suspected or confirmed diagnosis and initial or secondary signs of clinical severity (dyspnea, desaturation, etc.) under hospital care. |  |  |  |  |
